# Refugee and Migrant Children’s Mental Healthcare: Serving the Voiceless, Invisible, and the Vulnerable Global Citizens

**DOI:** 10.7759/cureus.9944

**Published:** 2020-08-22

**Authors:** Farah Khan, Noha Eskander, Therese Limbana, Zainab Salman, Parveez A Siddiqui, Syed Hussaini

**Affiliations:** 1 Psychiatry, California Institute of Behavioral Neurosciences & Psychology, Fairfield, USA; 2 Internal Medicine, Dubai Medical College, Dubai, ARE; 3 Internal Medicine, Stat Cardiologist, Chicago, USA; 4 Medicine, Windsor University School of Medicine, Basseterre, KNA

**Keywords:** refugee, migrant, mental health, asylum-seekers, trauma

## Abstract

Millions of children are on the run worldwide, with many unaccompanied children and adolescents undertaking risky journeys to flee war, adverse circumstances, and political persecution. The grueling journey and multiple stressors faced by the refugee children, both accompanied and unaccompanied during the pre-migration, migration, and in the country of destination, increase their risk for psychiatric disorders and other medical conditions. Unaccompanied refugee migrant children have higher prevalence of mental health disorders than accompanied refugee peers. Long after reaching the host country, the refugee, migrant, and asylum-seeking juveniles continue to face adversities in the form of acculturation. In assessing medical fitness and healthcare mediations for refugees and migrant children, special consideration should be given to certain areas such as their distinct history, whether they are with their family or separated or unaccompanied, and whether they have been peddled or have been left behind.

## Introduction and background

An alarming number of children travel with family or alone without proper care to flee organized violence, war, and persecution in their native country. Some cross their national borders to become refugees and seek asylum in other countries, a legal process recognized by the United Nations. Without a family or an adult, these children are often at risk of being exploited and abused. Most of the refugee children live in nearby countries close to their own native place of origin that happen to be low- or middle-income countries [1].

In 2016, in Italy about 92% of children arriving by sea were separated and unaccompanied [2]. In 2017, the high-income countries resettled 102,800 refugees [3]. In 2018, juveniles under 18 years of age incorporated about half of the refugee population [4]. In 2018, Uganda recorded 41,200 child refugees, the largest number of unaccompanied and separated child refugees with the overwhelming majority aged under 15 and a couple thousand aged under 5. From 2012-2018, Turkey has been hosting 3.7 million refugees, the largest refugee population [4]. In 2019, the most common country of origin among child asylum seekers happens to be Syrian Arab Republic. In 2019, Germany registered 35% of all child asylum applications lodged in Europe (71,420 children), while the highest number of first-time applicants with regard to its population was Greece [5].

The grueling journey faced by the refugee children, both accompanied and unaccompanied during the pre-migration, migration, and in the country of destination, is associated with multiple stressors resulting in elevated risks for psychiatric disorders and other medical conditions [6]. The clinicians should be aware that exposure to war, a long arduous journey with minimum or no care, and ongoing stressors that refugee kids have experienced are associated with physical, developmental, and mental health problems. This migration is in itself dangerous, and apart from mental and other health costs to it there is also an increased risk of disabilities, and vulnerabilities to acute and chronic ailments. This article discusses some of the commonly seen mental health conditions and other medical conditions in refugee and migrant children from the reviewed articles. It also provides an insight into the refugee mental health struggles during the coronavirus (COVID-19) pandemic and the migrant detention facilities.

## Review

Methods

Articles were obtained from PubMed using regular keywords and filters. Data and statistical information were also collected from United Nations High Commissioner for Refugees (UNHCR), a UN agency that helps refugees; the United Nations International Children’s Emergency Fund (UNICEF); and the World Health Organization (WHO) Regional Office for Europe.

PubMed Database

Regular keywords to search the PubMed database can be seen in Table 1.

**Table 1:** Keywords used to query the PubMed database

Keywords
Refugee
Mental health
Mental stress
Migrants
Asylum seekers

Studies were selected and reviewed after applying the inclusion/exclusion criteria on PubMed. The following were the inclusion criteria: (1) age 18 years and younger, (2) both female and male, (3) articles in English, and (4) studies published within the last one year. Exclusion criteria were age above 18 years and non-English articles.

The articles selected from PubMed were broken down as seen in Table 2.

**Table 2:** Article breakdown by type and number

Record	Number
Total records	51
Articles selected	35
Number of full-text articles	15
Abstracts only	20
Articles removed	16
Duplicates	0

Results

PubMed Database

**Figure 1:**
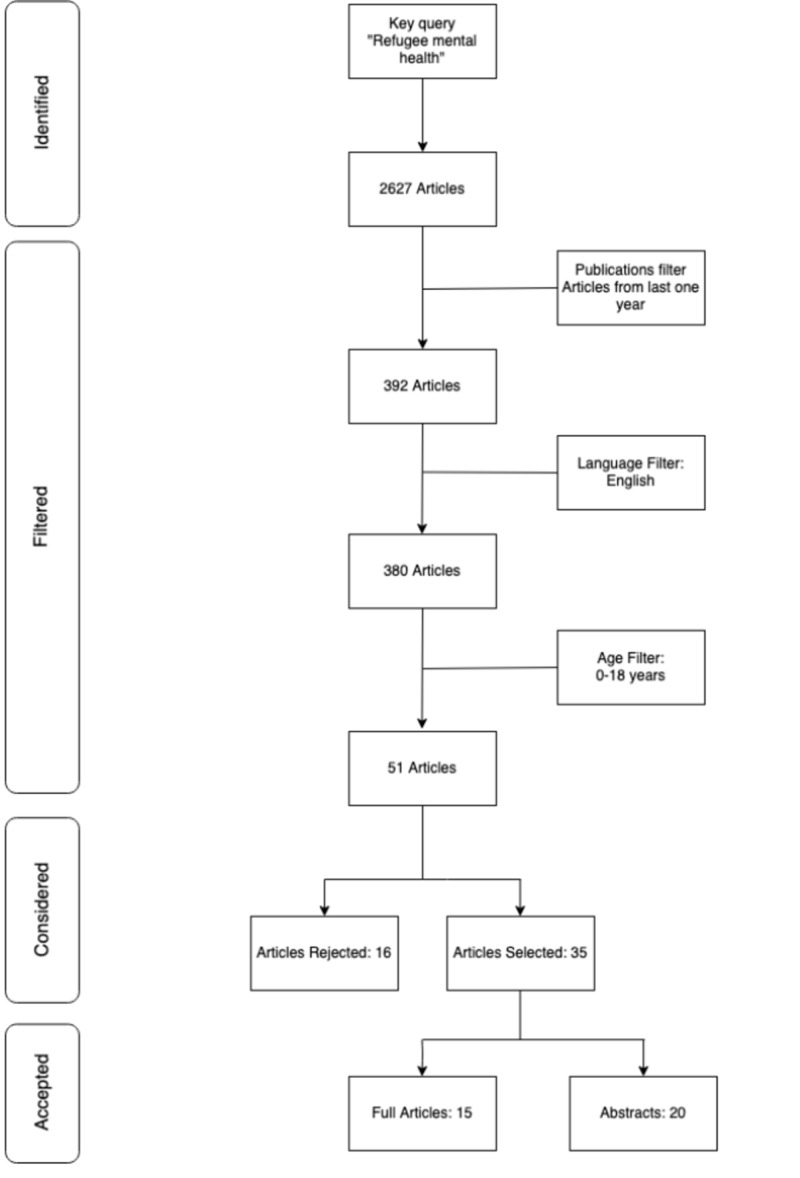
Screening and selection process for article review

After applying inclusion/exclusion criteria and using regular keywords, the total number of articles selected after review and refined search were 35 as they fit the selection criteria. The articles removed were not included for lack of relevant data. The flowchart seen in Figure 1 shows the starting keywords used, and the number of articles obtained on PubMed for literature search with the applied filters. Finally, the total number of used articles is displayed alongside those which were not selected.

Discussion

There are many facets of mental health for refugee children and adolescents that need to be addressed as they influence all potential determinants of health - including the societal culture of country of origin of the family, the journey to the host country, and living conditions in the host country [7]. The obstacles in the host country include education challenges, language barriers, economic opportunities, lack of understanding of the healthcare system, knowledge about available resources, issues pertaining to accessing health and other services, trust factor, financial problems, transportation issues, and the larger policy and political context of local authorities [7,8]. Primary care physicians, pediatricians, and mental health providers can build trust through culturally competent and trauma-informed care, assess for healthcare needs, provide vaccination update and preventative care, and screen for mental health, communicable diseases, disabilities, and other medical health conditions thereby attending to the holistic needs of the vulnerable child and adolescent refugees. Refugee children are less likely to avail pertinent health and social care than non-refugee children peers [9]. Most host countries offer some kind of health screening for refugees, both child and adult, upon entering the country of destination [3]. In assessing medical fitness and healthcare mediations for refugees and migrant children, special consideration should be given to certain areas such as their distinct history, whether they have migrated along with their family or have been separated from family, are unaccompanied, whether they have been peddled, or have been left behind [7]. Children’s right to medical care is guaranteed by all the world leaders and Member States of the WHO European Region and is compiled in the Convention on the Rights of the Child (CRC), a convention guaranteeing the highest attainable standard of healthcare and treatment of illness and rehabilitation of the refugee, migrant, and asylum-seeking children similar to the children native to the host country [3]. The most vulnerable children include the asylum seekers and the undocumented or unregistered migrants. Asylum seekers have usually been tested with war and/or political oppression in their native country and live in uncertainty and temporary circumstances regarding their future. The undocumented children often live in dangerous environments with little or no availability of basic societal rights, in abuse, poverty, brutality, and social boycott, and among threats of deportation [10].

Migration Risk Factors

Migrants face a myriad of issues during various aspects of their journey between countries. During the pre-migration phase of this process, there is a lack of access to health/dental care, scarcity of food, and exposure to diseases. During the journey, lack of access to health/dental care and food scarcity continue to be problems. Additionally, human trafficking, violence, and injuries during the trip are also present. Finally, in the country of destination, difficulty in finding resources presents itself as the largest barrier. These resources include health/dental care, education, therapy, and other basic amenities. This process repeats itself and becomes a cycle if migrants are deported back to their country of origin and seek to migrate once again [7].

Acculturation Stress

**Figure 2:**
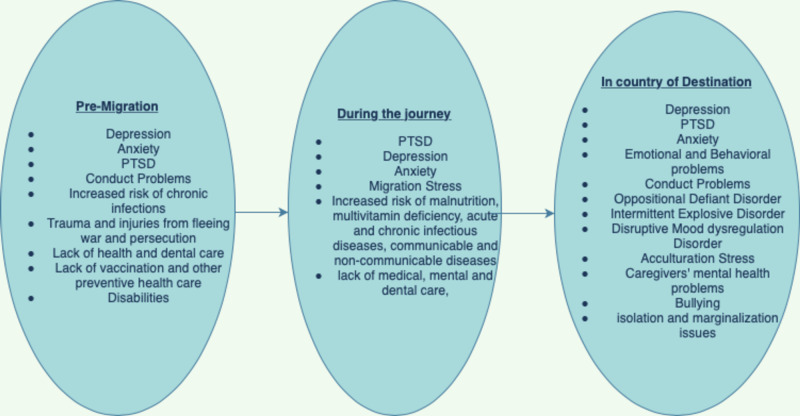
Mental and other health challenges of the refugee and migrant children from the reviewed articles PTSD, post-traumatic stress disorder

Refugees and migrants arriving in the host country, many of which have different cultures and languages from their native country, go through a course of learning and acclimatization to the new civilization. This stressful process of acculturation compounds the migration strain thereby amplifying the psychological distress. Children and adolescents, who are enrolled in school, generally learn the new host country language faster and conform to the new culture faster than parents, who may be secluded giving rise to new challenging family issues. Family tensions can cause disharmony, separation, and even assault, with associated adverse effects on a child’s mental health [11]. Some of the needs of the refugees and migrants include access to mental health services; the youths have a need for civil activities and community acceptance while the parental urgency is to feel culturally protected. Competency in a local language of the host country, and support from local community, school, and local authorities make their transitions easier and decrease the acculturation stress. Figure 2 shows some of the mental and other health challenges of the refugee and migrant children.

Health Issues

Communicable diseases: Cramped and overpopulated settlement and lack of cleanliness and sanitation in facilities housing refugee and migrant children put them at increased risk for diarrhea and skin infections [3]. The third-world countries show a higher prevalence of tuberculosis, malaria, intestinal parasites, and hepatitis B and C, than the developed nations. These chronic infections are present in increased prevalence in refugee and migrant juveniles [12,13]. A study reports of unaccompanied refugee and migrant children who were arriving in Germany with multidrug-resistant bacteria colonization at higher rates, and other records of a surge of measles, which is vaccine-preventable, have also been seen in asylum-seeking juveniles [14,15]. Clinicians should have a low threshold to screen for sexually transmitted infections (STIs) in adolescent refugees as they have the highest rate of curable STIs worldwide [16]. Some of the commonly seen non-communicable diseases include obesity and psychological problems in migrant children [17]. Obesity could be due to stress or change in dietary habits. Vitamin D deficiency is often caused by lack of exposure to sunshine in winter [18,19]. Other conditions like malnutrition and multivitamin deficiencies are most likely to be prevalent too due to lack of access to food and health care in the migration journey.

Mental Health

As per UNHCR data, about 138,600 of refugee children in 2018 were unaccompanied minors [4]. Segregation from parents can be harmful to a child’s health and prosperity, mostly mental health, as parents lay the foundation for the societal and environmental base for children [20]. Unaccompanied refugee minors have a higher prevalence of psychiatric disorders than accompanied refugee peers [21]. When accompanied by families, and after having experienced the migration trauma, children are often "hidden from sight" with no regard to their own personal wishes. Mental health must be seen as a complex primary healthcare need and should be served in a holistic and family-oriented manner whenever possible. Research studies have shown that freshly arrived migrant and refugee juveniles are at a high risk of psychosocial and mental issues due to exposure to organized crime and migration stress [22]. These are most commonly internalizing disorders - anxiety, depression, and post-traumatic stress disorder (PTSD) [23,24]. A study of asylum seekers with serious mental health problems, in the Netherlands, found that parental symptoms of PTSD were associated to infants’ troubled attachment and that parental apathy was related to parental PTSD [25]. A cohort study found that caregivers’ ordeal history and postmigration adversities were correlated with greater PTSD, rigid parenting, and an increase in child conduct problems [26]. Expressive symptoms, however, were found to be equal to that in children of the host country [24]. Longitudinal studies have shown that the high rate of internalizing symptoms tended to wear off slowly over a period of time, with expressions of PTSD fading away in about seven years after arrival to the host country [27]. Some frequently reported emotional and behavioral mental health problems among Bhutanese refugee youth include fighting, loneliness, depression, and being scared. Other symptoms of oppositional defiant disorder, intermittent explosive disorder, conduct disorder, generalized anxiety disorder, major depressive disorder, and disruptive mood dysregulation disorder were also seen among them [28]. Migrant and refugee juveniles frequently have to compromise more when parents are suffering from psychiatric disorders after dreadful experiences and migration strain. Parents with mental health challenges battle to give their children a feeling of support and stability [29]. Migration stress with socioeconomic deprivation takes a toll on the parents and increases the risk for child abuse [30]. Early and adequate cognitive, mental, and emotional support for parents suffering from behavioral disorders is thus a vital support for children. Refugees may hesitate to seek mental health help due to a culturally based stigma around mental health issues [9]. Family separation and parental death drives adolescents to take on parental roles for younger siblings. Recognition of these roles will enable physicians to provide suitable emotional and social support. Risk factors in the host country, such as financial hardships, parental separation, and aggression/bullying, were analyzed as vital determinants of mental health at follow-up [27]. In recent years, cognitive behavioral psychotherapy, eye movement desensitization and reprocessing (EMDR), and narrative exposure therapy for migrant and refugee children who have experienced war and displacement have been established for the evaluation and treatment of PTSD and depression [11]. Strengths of refugee children include personal resilience, parental support, close-knit family structure, and lasting association with their religious and cultural identity from the country of origin [31].

Coronavirus Pandemic and Mental Health

The staggering majority, 84%, of the global refugee population is accepted in developing regions with limited access to quality mental health even before the pandemic [32]. Now, they are overwhelmed with mental health crisis, as warned by the UNHCR. While many refugees and internally displaced people are exceptionally resilient, their abilities to cope are now being stretched to the limit. The loss of daily wages and livelihoods is taking a toll on their mental health and causing psychosocial hardships. Social distancing measures and limited mobility are compounding emotional distress with reports of self-harm increasing among the refugees. The COVID-19 precautions and reduced staffing levels during this pandemic are also impacting the availability of aid and mental health support as refugees are often unable to travel, and many face-to-face activities have been cancelled. The UNHCR is stepping up efforts to ensure the continuity of care by providing mental health services remotely through multi-lingual telephone hotlines and over the internet through online sessions. In addition, they are ensuring that people who need medication can continue treatment during lockdown [33].

Interventions

For Children 

The Teaching Recovery Techniques approach is used to decrease children’s discomfort and post-traumatic symptoms and to improve peer and kinfolk relations [34]. This psychosocial intervention is meant for juveniles who have experienced dreadful circumstances. Children are assembled in organized groups focused at augmenting emotional management, survival competency, and conflict resolution skills. These techniques also help the children to express themselves. There is also a parent component session to educate about intervention and on skills to reinforce care of their children.

For Parents 

Ladnaan intervention is a culturally adapted parenting guidance program combined with local civic orientation for Somali-born parents living in Sweden. A trained community educator of Somali origin facilitates the program. Parents report higher success and satisfaction after completing the course and convincing improvement in behavioral problems in their children. In this 12-week session, parents are educated on local community information, receive lectures and take part in workshops, and exchange views on the parent-child liaison, attachment, child growth, and development of interpersonal skills [35].

Mind-Spring is a mental health disorder prohibition plan in Belgium, Denmark, and the Netherlands. It provides psycho-education, and psychosocial and parenting skills for refugee and asylum-seeking parents in a culturally conscious manner in their own native language [3]. It deals with topics on mental health such as stress, ordeal, depression, personality, acculturation, and mental health fitness. The program promotes exchanging thoughts on experiences and provides parents with information about mental health expertise to recognize signs of suffering and mental ailment in themselves. It also educates the parents about obtaining help if and when needed. Parents also obtain the required skills and support in the parenting process and how to ploy collateral parenting issues.

School-Based Programs

Studies have shown that educational institutions play a vital role in conserving and promoting the health and well-being of refugee and migrant children. Successful school-based mental health prevention requires experts trained in cultural proficiency, who can interpret the mental health requirements and risks of refugee and migrant children, and who can conform the learning program to the needs of the individual child and family [34,36]. Hearing All Voices was a pilot project undertaken by Child to Child in London aimed at promoting social inclusion, commitment in education, and local community involvement among vulnerable youth, with a prime focus on refugee, migrant, and asylum-seeking youth [37]. The Pharos School Prevention Program conducts classroom-based program in the Netherlands with the aim of developing social involvement among migrant children with local host community children and adults while simultaneously attending to individualized requirements of each child [38]. Health assessments are performed for refugee and migrant children in a school setting in Malmo City, Sweden. Here, the school nurse meets the juveniles and their caregivers for a health assessment to define and address each child’s healthcare requirements. An analytical interview is followed by a broad general examination of the body, including dentition, eyesight, and hearing. Mental health is briefly assessed and vaccination history is analyzed. Referrals are made based on the necessity of specialized services.

Policy Consideration

National governments have a significant role in establishing living circumstances for refugee and migrant children as most freshly settled refugee families rely on national and local authorities’ support for habitation and existential expenses. Governments determine the rights of children to access health care maintenance and educational benefits in their country. Policies that exemplify humanity should be planned and implemented for the refugees/migrants and asylum seekers.

Health Advocacy

A detailed individualized health evaluation by a healthcare professional on arrival to the host country should determine the healthcare needs and screen for communicable diseases; disability should be assessed and vaccinations should be updated. This response will help detect infections early on, allow timely treatment to be given, and will be most cost effective in the long run. The availability of medical translators and native cultural arbitrators is important to ensure the best healthcare outcome for refugee and migrant children. Blueprints to improve welfare, and access to education and health in refugee and migrant children should have a comprehensive framework that targets risk factors on individual, family, and community levels. Culturally sensitive, parent and other caregiver support curriculum and interventions in the school and local community centers should be promoted. Transferring children between multiple locations should be minimized as it disrupts the peer networks and educational flow; this also holds good for unaccompanied children with substitute caregivers. In order to build good relations with substitute caregivers, unaccompanied children need consistent long-term, definitive housing with the same guardians. The most vital physical, social, and psychological support for children are their parents; therefore, family reunions should be expedited [7].

Detention of Migrant Children

The United States of America has built the largest immigration detention system in the world. In 2019, a staggering 62,550 migrant children including infants, toddlers, kids, and teens were held in custody in facilities across USA. These facilities lack enough clinicians or specialized care for the detained children [39]. Immigration detention has adverse and detrimental consequences for the well-being of those detained, but studies have found that it is most inimical to children [40]. The negative impacts of detention on mental health are more brutal for children than for adults; therefore, detention should not be weaponized for deportation of migrant children. If this is inevitable, then the facilities harboring children should have child-friendly areas, and avenues for healthcare and education should be provided. Children on the move also suffer brutality, injustice, and misconduct from law enforcement officials - local police, border guards, and detention officers. Such events cause children to quickly learn to mistrust authorities. These adverse psychological effects may last years after release from detention [41].

## Conclusions

Children are global citizens and their rights move with them; therefore, their healthcare needs should not be defined by geographic borders. Mounting evidence suggests welcoming and supportive policies for refugee, migrant, and asylum-seeking children can prevent psychological distress and mental health disorders in these vulnerable children. All-inclusive policies that aim at protecting the rights of every child should be enforced globally. Children should not be held in detention centers indefinitely in subhuman conditions away from their parents/primary caregivers. Reuniting children with their families should be prioritized and expedited. It is imperative to enforce preventative mental health policies and refrain from practices that abuse human rights. Healthcare providers should consider volunteering in refugee and migrant camps, and also in local community free clinics that are accessed by refugee and migrant children. This will ensure adequate staffing in detention facilities specially during the pandemic where the invisible, voiceless, and vulnerable refugee and migrant children along with the adult refugees and migrants can get timely medical attention and treatment for their healthcare needs. Research is needed on improving resilience building and for appraising the impact of precise interventions that could improve outcomes. More longitudinal studies are needed to assess interventions that increase better mental health outcomes, community inclusion, better educational results, and increase job opportunities.
